# First-in-Human Dose-Escalation Study of Fianlimab, an Antilymphocyte Activation Gene-3 Antibody, with Cemiplimab in Patients with Advanced Malignancies

**DOI:** 10.1158/1078-0432.CCR-23-3883

**Published:** 2024-10-18

**Authors:** Nehal J. Lakhani, Kyriakos P. Papadopoulos, Melissa Lynne Johnson, Haeseong Park, Ding Wang, Timothy A. Yap, Afshin Dowlati, Robert G. Maki, Susanna Ulahannan, Filipa Lynce, Karen Kelly, Stephen Williamson, Jyoti Malhotra, Shuquan Chen, Ana Gonzalez Ortiz, Vladimir Jankovic, Anne Paccaly, Sheila Masinde, Jayakumar Mani, Israel Lowy, Giuseppe Gullo, Tasha Sims, Glenn Kroog

**Affiliations:** 1 START Midwest, Grand Rapids, Michigan.; 2 START San Antonio, San Antonio, Texas.; 3 Sarah Cannon Research Institute, Tennessee Oncology, PLLC, Nashville, Tennessee.; 4 Washington University School of Medicine, St. Louis, Missouri.; 5 Henry Ford Hospital, Detroit, Michigan.; 6 University of Texas MD Anderson Cancer Center, Houston, Texas.; 7 Case Comprehensive Cancer Center, Case Western Reserve University, Cleveland, Ohio.; 8 Cold Spring Harbor Laboratory, Cold Spring Harbor, New York.; 9 Abramson Cancer Center, University of Pennsylvania Perelman School of Medicine, Philadelphia, Pennsylvania.; 10 Stephenson Cancer Center, University of Oklahoma Health Sciences Center, Oklahoma City, Oklahoma.; 11 Lombardi Comprehensive Cancer Center, MedStar Georgetown University Hospital, Washington, DC.; 12 UC Davis Comprehensive Cancer Center, UC Davis Health, Sacramento, California.; 13 University of Kansas Medical Center, Fairway, Kansas.; 14 Rutgers Cancer Institute of New Jersey, New Brunswick, New Jersey.; 15 Regeneron Pharmaceuticals, Inc., Tarrytown, New York.

## Abstract

**Purpose:**

Preclinical data indicate that fianlimab (antilymphocyte activation gene-3) plus cemiplimab (anti–PD-1) enhances antitumor activity. Here, we report prespecified final analyses of the dose-escalation part of a first-in-human, phase 1 study (NCT03005782) of fianlimab as monotherapy and in combination with cemiplimab in patients with advanced malignancies.

**Patients and Methods:**

Adult patients received 1 to 40 mg/kg of fianlimab plus 350 mg of cemiplimab every 3 weeks (Q3W) across various dose-escalation schedules. Primary objectives were the rate of dose-limiting toxicities, adverse events (including immune mediated), deaths, laboratory abnormalities, and pharmacokinetics. Secondary outcomes were objective response rate, best overall response, duration of response, and antidrug antibody variables.

**Results:**

Seventy-eight patients were enrolled (fianlimab + cemiplimab, *n* = 47; fianlimab monotherapy, *n* = 31). One patient treated with 3 mg/kg fianlimab + cemiplimab experienced dose-limiting toxicities, including increased blood creatine phosphokinase and myasthenic syndrome. No maximum tolerated dose was reached. Any-grade treatment-emergent adverse events occurred in 90% of patients with fianlimab monotherapy, in 87% of patients with fianlimab + cemiplimab, and in 87% of patients who transitioned from monotherapy to combination therapy. Fianlimab pharmacokinetics were dose proportional and similar in monotherapy and combination therapy. Across patients who received fianlimab + cemiplimab, five achieved a partial response, three of whom experienced a response after transitioning from monotherapy to combination therapy. Fianlimab 1,600 mg Q3W (20 mg/kg in an 80-kg individual) is the selected dose for phase 2 and phase 3 studies.

**Conclusions:**

Fianlimab as monotherapy and in combination with cemiplimab demonstrated acceptable safety and preliminary antitumor activity, which is generally consistent with previous reports of cemiplimab.

**Translational Relevance**

In the rapidly evolving field of oncology therapies, a combination of antilymphocyte activation gene-3 (anti-LAG-3) plus anti-PD-1 (relatlimab plus nivolumab) recently demonstrated superior efficacy to anti-PD-1 monotherapy in a phase 3 study of patients with advanced melanoma, leading to the first regulatory approval of an anti-LAG-3 to treat human malignancies. Preclinical data suggest that fianlimab (anti-LAG-3) combined with cemiplimab (anti-PD-1) enhances cemiplimab antitumor activity; these data provide the rationale for exploring fianlimab plus cemiplimab in a first-in-human dose-escalation study in patients with advanced malignancies. Here, fianlimab monotherapy and fianlimab plus cemiplimab demonstrated an acceptable safety profile, consistent with previous reports for cemiplimab monotherapy; preliminary clinical activity suggests that fianlimab plus cemiplimab may enhance the antitumor activity of cemiplimab. Together, our findings support previous preclinical and clinical studies in providing evidence of a potential alternative anti-LAG-3 and anti-PD-1 combination therapy.

## Introduction

Lymphocyte activation gene-3 (LAG-3) is an immune checkpoint receptor that delivers an inhibitory signal to activated T cells upon major histocompatibility complex (MHC) class II binding, and which is upregulated on infiltrating T cells in many cancer types (1, 2). It has also been suggested that the secondary LAG-3 ligands galectin-3, liver and lymph node sinusoidal endothelial cell C-type lectin, fibrinogen-like protein 1, α-synuclein preformed fibrils, and the T-cell antigen receptor/CD3 complex mediate LAG-3 immunosuppressive functions (3). In preclinical models of cancer and chronic viral infection, it has been observed that the blockage of LAG-3 reverses T-cell exhaustion (4, 5). LAG-3 is coexpressed with PD-1 on tumor-infiltrating lymphocytes, and preclinical evidence suggests that dual blockade of LAG-3 and PD-1 may enhance antitumor immune activity (6). Fianlimab (REGN3767) and cemiplimab are both high-affinity, fully human, hinge-stabilized immunoglobulin G4 monoclonal antibodies (7). Fianlimab blocks LAG-3/MHC class II–driven T-cell inhibition. Cemiplimab is an anti-PD-1 monoclonal antibody that blocks the interactions of PD-1 with PD-L1 and PD-L2 (7, 8). Cemiplimab is approved for the treatment of certain patients with advanced cutaneous squamous cell carcinoma, for patients with locally advanced or metastatic basal cell carcinoma, for the first-line treatment of patients with advanced non–small cell lung cancer with high PD-L1 expression and no *EGFR*, *ALK*, or *ROS1* aberrations, and in combination with platinum-based chemotherapy for the first-line treatment of patients with advanced non–small cell lung cancer with no *EGFR*, *ALK*, or *ROS1* aberrations (9).

In humanized PD-1 and LAG-3 knockin mice, in which the extracellular domains of mouse *Pdcd1* and *LAG-3* were replaced with their human counterparts, treatment with fianlimab improved the antitumor immune efficacy of cemiplimab, with enhanced secretion of proinflammatory cytokines by tumor-specific T cells (10). These data provided the rationale for exploring fianlimab in combination with cemiplimab in this phase 1 trial, further supported by recent results showing improved median progression-free survival and objective response rate (ORR) with combined nivolumab (anti-PD-1) and relatlimab (anti-LAG-3) compared with anti-PD-1 monotherapy in patients with treatment-naïve advanced melanoma (11).

Here we report the safety, pharmacokinetics, and preliminary antitumor activity of fianlimab as monotherapy or in combination with cemiplimab, as well as the selection of the recommended phase 2 dose from the final analysis of the dose-escalation part of this study.

## Patients and Methods

### Study design and participants

This phase 1, first-in-human, open-label, multicenter study (ClinicalTrials.gov identifier NCT03005782; EudraCT number 2016-002789-30) was composed of two parts: a dose-escalation part and a cohort-expansion part using the recommended phase 2 dose. The focus of this publication is the dose-escalation part, the primary objective of which was to determine the safety, pharmacokinetics, and selection of the recommended phase 2 dose of fianlimab for the expansion cohorts. The data cutoff date was August 25, 2021.

The dose-escalation part enrolled adult patients with advanced malignancies who did not respond to or showed progression despite standard therapy or who had incurable disease and for whom no available therapy was expected to convey clinical benefit. All patients were naïve to prior anti-LAG-3 therapy and all except three patients were naïve to prior anti-PD-1/PD-L1 therapy. Eligible tumor type was any solid tumor or lymphoma that was appropriate for a phase 1 first-in-human study. Additional inclusion criteria were an Eastern Cooperative Oncology Group performance status of ≤1, adequate organ and bone marrow function, and ≥1 radiographically measurable lesion per Response Evaluation Criteria in Solid Tumors version 1.1.

Key exclusion criteria were prior treatment with any LAG-3–targeting biologic or small molecule, ongoing or recent (within 5 years) autoimmune disease requiring systemic immunosuppression, and treatment with immunosuppressive doses of steroids (prednisone >10 mg daily or equivalent). Full inclusion and exclusion criteria are available in the study protocol.

The dose-escalation part of this study investigated fianlimab as monotherapy or in combination with cemiplimab in a staggered manner. Five planned dose levels of fianlimab [1, 3, 10, 20, and 40 mg/kg every 3 weeks (Q3W)] administered as a 30-minute intravenous infusion were investigated as monotherapy. Six dose levels of fianlimab in combination with cemiplimab, which was given as a 30-minute intravenous infusion after fianlimab, were investigated: fianlimab 1, 3, and 10 mg/kg Q3W in combination with cemiplimab 3 mg/kg Q3W, and fianlimab 10, 20, and 40 mg/kg Q3W in combination with cemiplimab 350 mg Q3W. The clinical dose of cemiplimab evolved from body weight–adjusted dosing to fixed dosing in analogy to other anti-PD-1 drugs and to match the FDA-approved dose of 350 mg Q3W (9).

A modified 3+3 design (4+3) was used to evaluate all dose levels in the fianlimab monotherapy and fianlimab-plus-cemiplimab cohorts, respectively (Supplementary Fig. S1). Four patients were enrolled at each dose level to minimize delay in case a patient discontinued before being evaluable for dose-limiting toxicities (DLTs). Dose level tolerability was evaluated over a 28-day DLT period.

Any patient who experienced progressive disease during treatment with fianlimab monotherapy but tolerated a minimum of two doses of fianlimab monotherapy was allowed to cross over to fianlimab at the highest dose found tolerable in the study at that point, in combination with cemiplimab 350 mg Q3W. Data collected from these patients during their monotherapy treatment are included in the monotherapy treatment category, and data collected during their re-treatment phase (fianlimab-plus-cemiplimab combination treatment) are presented separately as the monotherapy-to-combination-therapy subset. Duration of the treatment period was 51 weeks or until disease progression, unacceptable toxicity, withdrawal of consent, or withdrawal from the study, whichever came first. After 51 weeks, patients may have received an additional 51 weeks of treatment at the discretion of the investigator, provided that the patient had experienced clinical benefits.

### Procedures

The safety and tolerability of fianlimab as monotherapy or in combination with cemiplimab was monitored by clinical assessment of adverse events as well as evaluations of vital signs, physical examinations, 12-lead electrocardiograms, and laboratory assessments including standard hematology, chemistry, and urinalysis, with data analyzed by treatment dose and combination. Adverse events and laboratory abnormalities were graded according to the NCI's Common Terminology Criteria for Adverse Events version 5.

Blood samples were collected to determine concentrations of fianlimab and cemiplimab via ELISA and to assess immunogenicity [antidrug antibodies (ADA)] via an electrochemiluminescence bridging immunoassay in serum over time. Fianlimab and cemiplimab concentrations in the serum were analyzed using a lower limit of quantitation of 0.078 mg/L in undiluted human serum.

Peripheral blood mononuclear cell (PBMC) samples were collected for additional biomarker analysis and were used for longitudinal assessments of T-cell proliferation and activation in circulation using flow cytometry. Briefly, PBMCs were isolated from whole peripheral blood samples using Ficoll gradient extraction and cryopreserved for flow cytometry analysis. Multiparameter flow cytometry assay was performed on batched PBMC samples collected at baseline and after treatment over the first two cycles of therapy in the fianlimab monotherapy and cemiplimab combination dose-escalation cohorts. The flow cytometry assay included cell surface markers of effector memory T-cell subsets (CD4, CD8, CCR7, CD45RA, PD-1), T-cell proliferation (Ki67), and T-cell activation (HLA-DR). Tumor immunohistochemistry for detection of LAG-3 was performed in formalin-fixed, paraffin-embedded tissues using a mouse antibody clone (17B4, Abcam) developed and validated using the OptiView detection and amplification kit systems (Ventana Medical Systems, Inc.)

Antitumor activity (ORR) was assessed by CT and MRI, or by PET-CT only for patients with lymphoma. Imaging tumor assessments were performed every 42 days (±7 days) for the first 168 days, then every 63 days from day 169 through day 357. The full assessment schedule is available in the study protocol.

### Objectives

The primary study objectives were the rate of DLTs, defined as study drug toxicity that leads to inability to administer the second dose of the study drug within 35 days of cycle 1 day 1; any grade ≥3 nonhematologic toxicity (excluding clinically insignificant laboratory abnormalities such as asymptomatic elevations in amylase or lipase), grade ≥2 uveitis, grade 4 neutropenia lasting >7 days or thrombocytopenia, grade 3 thrombocytopenia with bleeding, and grade ≥3 febrile neutropenia or neutropenia with documented infection within the 28-day treatment window; and pharmacokinetics including drug concentrations of fianlimab and cemiplimab in the serum. Immune-mediated adverse events (imAE) were defined as any adverse event thought to be caused by unrestrained immune responses directed at normal host tissue.

Key secondary objectives included ORR, defined as the number of patients with the best overall response of confirmed complete response or partial response; the best overall response, defined as the best response recorded from the start of the treatment until disease progression or recurrence; the duration of response, defined as the time between the first measurement of confirmed complete response or partial response and the first date of recurrent or progressive disease or death; and ADA variables, including the presence of ADAs, treatment-emergent ADAs to fianlimab, and titer level. An exploratory objective was pharmacodynamic longitudinal assessment of activated T-cell populations in circulation.

### Trial oversight

The study protocol and all amendments were approved by the appropriate institutional review board or independent ethics committee at each participating study site. The study was conducted in accordance with the principles of the Declaration of Helsinki and the International Conference on Harmonization Good Clinical Practice guidelines. All patients provided written informed consent before enrollment. Further details are available in the study protocol.

### Statistical analysis

There was no formal statistical hypothesis for the dose-escalation part of the study; analyses were prespecified and descriptive. All clinical safety and efficacy outcomes were analyzed using the safety analysis set, which included all patients who received any dose of study treatment. Up to 89 patients evaluable for DLTs were planned based on the modified 3+3 (4+3) design for each dose-escalation cohort.

### Data availability

Qualified researchers may request access to study documents (including the clinical study report, study protocol with any amendments, blank case report form, and statistical analysis plan) that support the methods and findings reported in this article. Individual anonymized participant data will be considered for sharing once the product and indication have been approved by major health authorities (e.g., FDA, European Medicines Agency, Product Development and Management Association, etc.) and if there is legal authority to share the data and there is not a reasonable likelihood of participant reidentification. Submit requests to https://search.vivli.org/enquiries.

## Results

### Patients and treatments

Between November 2016 and August 2019, 78 patients were enrolled: 47 received fianlimab plus cemiplimab and 31 received fianlimab monotherapy. Of the 31 patients who initially received fianlimab monotherapy, 16 transitioned at disease progression from fianlimab monotherapy to fianlimab-plus-cemiplimab combination therapy.

Baseline patient characteristics are summarized in Table 1. The median age (interquartile range) was 68.0 (59.0–74.0) years among patients who received fianlimab monotherapy, 60.0 (49.0–67.0) years in patients who received fianlimab plus cemiplimab, and 63.0 (55.5–72.5) years in the monotherapy-to-combination-therapy subset. Eastern Cooperative Oncology Group performance status was 1 in most patients, and most patients had previously received ≥3 systemic therapies.

**Table 1.** Baseline characteristics and prior therapies.

Characteristic	Fianlimab monotherapy (*n* = 31)	Fianlimab + cemiplimab (*n* = 47)	Monotherapy to combination^a^ (*n* = 16)
Age, median (range), years	68.0 (22–83)	60.0 (30–83)	63.0 (22–83)
Female, *n* (%)	17 (54.8)	27 (57.4)	9 (56.3)
ECOG performance status, *n* (%)			
0	6 (19.4)	16 (34.0)	5 (31.3)^b^
1	25 (80.6)	31 (66.0)	11 (68.8)^b^
Prior lines of systemic therapy, *n* (%)			
Any	29 (93.5)	46 (97.9)	15 (93.8)^c^
1	6 (19.4)	10 (21.3)	5 (31.3)^c^
2	5 (16.1)	7 (14.9)	4 (25.0)^c^
3	7 (22.6)	9 (19.1)	2 (12.5)^c^
4	1 (3.2)	8 (17.0)	0^c^
≥5	10 (32.2)	12 (25.5)	4 (25.0)^c^
Prior radiotherapy, *n* (%)	18 (58.1)	30 (63.8)	11 (68.8)

a A subset of patients who received fianlimab monotherapy as primary treatment, then subsequently received fianlimab-plus-cemiplimab combination therapy during a re-treatment phase. Data presented were gathered from the period during which patients were receiving combination therapy.

b ECOG performance status was at the time of starting fianlimab monotherapy.

c Number of prior lines of systemic therapy did not include fianlimab monotherapy before combination therapy.

Abbreviation: ECOG, the Eastern Cooperative Oncology Group.

The most common tumor type in the total patient population was colon cancer, reported in three (10%) patients in the monotherapy group and 11 (23%) patients in the combination therapy group (Supplementary Table S1).

At the time of data cutoff (August 25, 2021), 30 (97%) patients who received fianlimab monotherapy, 47 (100%) patients who received combination therapy, and 16 (100%) patients in the monotherapy-to-combination-therapy subset had discontinued treatment. The primary reason for discontinuation in all treatment arms was disease progression (Supplementary Table S2). One (3%) patient in the fianlimab monotherapy group completed the treatment period. The median duration of treatment exposure was 11.3 weeks among patients who received fianlimab monotherapy, 9.0 weeks among patients who received combination therapy, and 15.1 weeks among those who transitioned from monotherapy to combination therapy (Supplementary Table S3).

### Safety

No patient experienced a treatment-emergent adverse event (TEAE) leading to dose reduction in any treatment group. Four (13%) patients who received fianlimab monotherapy experienced a TEAE leading to a dose delay, and one (3%) patient experienced a TEAE leading to interruption of infusion. With fianlimab-plus-cemiplimab combination therapy, TEAEs leading to a dose delay or interruption of infusion occurred in seven (15%) and three (6%) patients, respectively. In the monotherapy-to-combination-therapy subset, TEAEs leading to a dose delay or interruption in infusion occurred in five (31%) patients and one (6%) patient, respectively.

No DLTs were observed in patients treated with fianlimab monotherapy up to and including the maximum administered dose of 40 mg/kg. One (2%) patient who received combination therapy experienced DLTs of increased blood creatine phosphokinase (grade 4) associated with myasthenic syndrome (grade 3) and increased troponin (grade 1) at the fianlimab 3 mg/kg + cemiplimab 3 mg/kg Q3W dose level. The protocol-defined DLT did not impact dose escalation. Because the DLT observation period was 28 days from the first assigned dose, DLTs were not formally investigated during the re-treatment phase of the monotherapy-to-combination-therapy subset.

TEAEs of any grade occurred in 28 (90%) patients in the monotherapy group; 12 (39%) patients experienced a grade ≥3 TEAE (Table 2; Supplementary Table S4). The most common TEAEs among patients who received fianlimab monotherapy were nausea (23%), abdominal pain (19%), and decreased appetite (19%). Increasing dose levels of fianlimab monotherapy did not seem to significantly impact the rate of TEAEs (Supplementary Table S5). In the combination therapy group, TEAEs of any grade occurred in 41 (87%) patients; 22 (47%) patients experienced a grade ≥3 TEAE (Table 2; Supplementary Table S4). The most common TEAEs were fatigue (36%), nausea (21%), and decreased appetite, diarrhea, and vomiting (all 17%). Increasing dose levels of fianlimab in combination with cemiplimab did not seem to correlate with the rate of TEAEs (Supplementary Table S5). In the monotherapy-to-combination-therapy subset, TEAEs of any grade occurred in 14 (88%) patients; six (38%) patients experienced a grade ≥3 TEAE (Table 2; Supplementary Table S4). The most common TEAEs were fatigue (44%), maculopapular rash (31%), and diarrhea, nausea, peripheral edema, and decreased appetite (all 25%).

**Table 2.** Summary of TEAE regardless of attribution.

*n* (%)	Fianlimab monotherapy (*n* = 31)		Fianlimab + cemiplimab (*n* = 47)		Monotherapy to combination^a^ (*n* = 16)	
	Any grade	Grade ≥3	Any grade	Grade ≥3	Any grade	Grade ≥3
Any	28 (90.3)	12 (38.7)	41 (87.2)	22 (46.8)	14 (87.5)	6 (37.5)
Serious	6 (19.4)	5 (16.1)	7 (14.9)	7 (14.9)	3 (18.8)	3 (18.8)
Led to discontinuation	0	0	0	0	2 (12.5)	2 (12.5)
With an outcome of death	1 (3.2)	1 (3.2)	1 (2.1)	1 (2.1)	0	0
Events that occurred in >10% of patients in any group, ordered by frequency in patients who received fianlimab monotherapy						
Nausea	7 (22.6)	1 (3.2)	10 (21.3)	1 (2.1)	4 (25.0)	1 (6.3)
Abdominal pain	6 (19.4)	0	2 (4.3)	0	1 (6.3)	0
Decreased appetite	6 (19.4)	0	8 (17.0)	2 (4.3)	4 (25.0)	1 (6.3)
Cystitis	0	0	5 (10.6)	0	1 (6.3)	1 (6.3)
Diarrhea	5 (16.1)	0	8 (17.0)	0	4 (25.0)	0
Fatigue	5 (16.1)	0	17 (36.2)	1 (2.1)	7 (43.8)	0
Vomiting	5 (16.1)	1 (3.2)	8 (17.0)	1 (2.1)	3 (18.8)	0
Anemia	4 (12.9)	3 (9.7)	7 (14.9)	3 (6.4)	0	0
Back pain	4 (12.9)	0	1 (2.1)	0	0	0
Dyspnea	4 (12.9)	1 (3.2)	3 (6.4)	2 (4.3)	0	0
Nasal congestion	4 (12.9)	0	1 (2.1)	0	1 (6.3)	0
Constipation	3 (9.7)	0	6 (12.8)	0	0	0
Cough	3 (9.7)	0	5 (10.6)	1 (2.1)	2 (12.5)	0
Headache	3 (9.7)	0	7 (14.9)	0	2 (12.5)	0
Hypotension	3 (9.7)	0	2 (4.3)	1 (2.1)	2 (12.5)	0
Infusion-related reaction	3 (9.7)	0	7 (14.9)	0	2 (12.5)	0
Abdominal distension	2 (6.5)	0	1 (2.1)	0	2 (12.5)	0
Dehydration	2 (6.5)	0	2 (4.3)	2 (4.3)	2 (12.5)	0
Edema peripheral	2 (6.5)	0	5 (10.6)	0	4 (25.0)	0
Hypokalemia	2 (6.5)	0	5 (10.6)	1 (2.1)	2 (12.5)	2 (12.5)
Hyponatremia	2 (6.5)	1 (3.2)	5 (10.6)	3 (6.4)	1 (6.3)	1 (6.3)
Tumor pain	2 (6.5)	0	5 (10.6)	0	0	0
Chills	1 (3.2)	0	7 (14.9)	0	0	0
Pyrexia	1 (3.2)	0	6 (12.8)	0	0	0
Rash maculopapular	1 (3.2)	0	1 (2.1)	0	5 (31.3)	2 (12.5)
Urinary tract infection	1 (3.2)	0	6 (12.8)	3 (6.4)	0	0
Arthralgia	0	0	3 (6.4)	0	3 (18.8)	0
Fall	0	0	1 (2.1)	0	2 (12.5)	0
Hypothyroidism	0	0	7 (14.9)	1 (2.1)	1 (6.3)	0
Skin infection	0	0	1 (2.1)	0	2 (12.5)	0

a A subset of patients who received fianlimab monotherapy as primary treatment, then subsequently received fianlimab-plus-cemiplimab combination therapy during a re-treatment phase. Data presented were gathered from the period during which patients were receiving combination therapy.

imAEs of any grade occurred in three (10%) patients in the monotherapy group; one (3%) patient experienced grade ≥3 imAEs of increased alanine aminotransferase and increased aspartate aminotransferase (Supplementary Table S6). imAEs of any grade occurred in 15 (32%) patients in the combination therapy group; three (6%) patients experienced a grade ≥3 imAE (Supplementary Table S6). The most common imAE of any grade was hypothyroidism (15%; *n* = 7). imAEs of any grade occurred in 10 (63%) patients in the monotherapy-to-combination therapy subset (Supplementary Table S6); the most common imAEs of any grade were diarrhea (25%; *n* = 4) and maculopapular rash (25%; *n* = 4).

Treatment-related adverse events (TRAE; as assessed by the investigators) of any grade occurred in 11 (35%) patients in the monotherapy group; two (6%) patients experienced a grade ≥3 TRAE (Supplementary Table S7). The most common TRAEs were infusion-related reaction (10%) and nausea (10%; Supplementary Table S7). In the combination therapy group, TRAEs of any grade occurred in 32 (68%) patients; five (11%) patients experienced a grade ≥3 TRAE (Supplementary Table S7). The most common TRAEs were fatigue (17%), hypothyroidism (15%), and infusion-related reaction (15%). Two (4%) patients experienced grade 2 adrenal insufficiency. Increasing dose levels of fianlimab in combination with cemiplimab did not seem to correlate with the rate of TRAEs. In the monotherapy-to-combination-therapy subset, TRAEs of any grade occurred in 11 (69%) patients; four (25%) patients experienced a grade ≥3 TRAE (Supplementary Table S7). The most common TRAEs were fatigue (38%; *n* = 6), diarrhea (25%; *n* = 4), and maculopapular rash (25%; *n* = 4). There were no treatment-related deaths in the dose-escalation phase of the study.

### Efficacy

The ORRs reported here are based on investigator assessment.

Among patients who received fianlimab monotherapy, there were no responses (Table 3). The best response of stable disease was achieved in 13 (42%) of these patients (Table 3; Supplementary Fig. S2).

**Table 3.** Investigator-assessed tumor response rate by Response Evaluation Criteria in Solid Tumors version 1.1.

*n* (%)	Fianlimab monotherapy (*n* = 31)	Fianlimab + cemiplimab (*n* = 47)	Monotherapy to combination^a^ (*n* = 16)
Objective response rate	0	2 (4.3)	3 (18.8)
Best overall response			
Complete response	0	0	0
Partial response	0	2 (4.3)	3 (18.8)
Stable disease	13 (41.9)	12 (25.5)	6 (37.5)
Progressive disease	10 (32.3)	26 (55.3)	4 (25.0)
Not evaluated	8 (25.8)	7 (14.9)	3 (18.8)
Disease control rate	13 (41.9)	14 (29.8)	9 (56.3)

a A subset of patients who received fianlimab monotherapy as primary treatment, then subsequently received fianlimab-plus-cemiplimab combination therapy during a re-treatment phase. Data presented were gathered from the period during which patients were receiving combination therapy.

**Figure 1.**
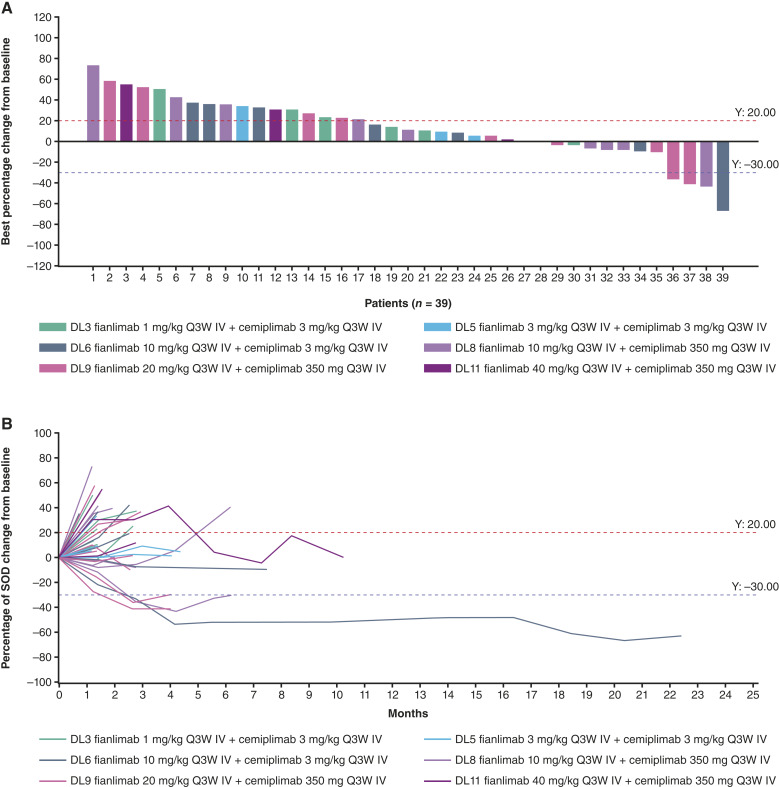
**A,** Clinical activity and (**B**) changes to target lesion over time in patients treated with fianlimab plus cemiplimab. Figure only includes patients who had both baseline and postbaseline target lesion assessments; not all patients had these assessments–therefore some patients may not be shown in this figure. Seven patients (7/47, 14.9%) were not evaluated in this treatment group. DL, dose level; IV, intravenous; Q3W, every 3 weeks; SOD, sum of diameters.

The ORR among patients who received fianlimab-plus-cemiplimab treatment was 4% (*n* = 2; 95% confidence interval, 0.5%–14.5%; Table 3). Both patients had small cell lung cancer and demonstrated partial response. The duration of response for these two responders was 3.5 and 19.7 months. Twelve (26%) patients had stable disease (Fig. 1; Table 3).

**Figure 2.**
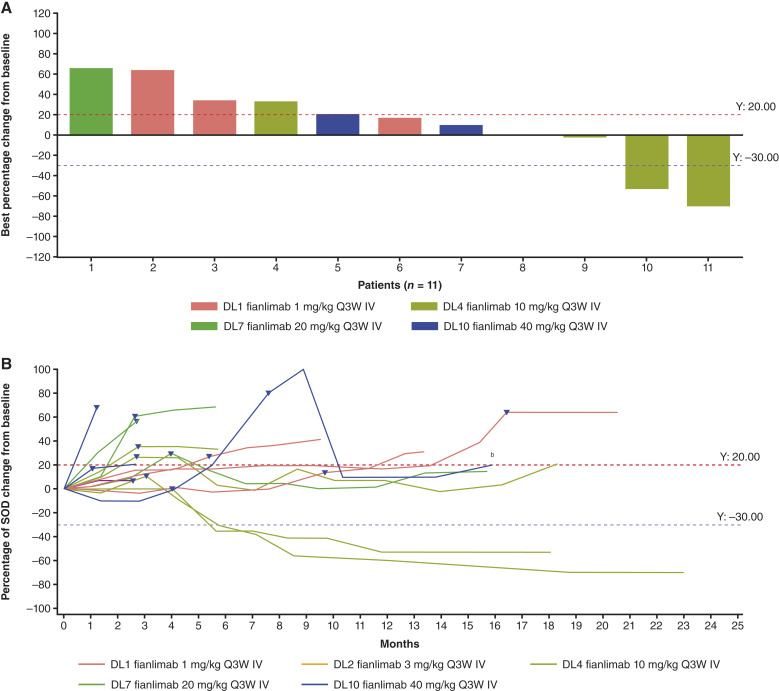
**A,** Clinical activity^a^ and (**B**) changes to target lesion over time in patients treated with monotherapy to combination therapy. ^a^Best overall response is calculated on the basis of tumor change from the start of combination treatment. ^b^Tumor response for this patient was calculated as the best change in tumor size from the start of combination therapy. Triangles denote the last tumor assessment on fianlimab monotherapy. Figure only includes patients who had both baseline and postbaseline target lesion assessments; not all patients had these assessments—therefore some patients may not be shown in this figure. Three patients (3/16, 18.8%) were not evaluated in this treatment group. DL, dose level; IV, intravenous; Q3W, every 3 weeks; SOD, sum of diameters.

In the monotherapy-to-combination-therapy subset, the ORR was 19% (*n* = 3; 95% confidence interval: 4.0%–45.6%); all three patients (endometrial cancer, *n* = 1; cutaneous squamous cell carcinoma, *n* = 1; and colon adenocarcinoma, *n* = 1) had a partial response (Table 3). Six (38%) patients had stable disease (Fig. 2). One patient with intrahepatic cholangiocarcinoma who had a partial response before disease progression on cemiplimab monotherapy (in a prior first-in-human study) experienced stable disease with tumor shrinkage during treatment with fianlimab-plus-cemiplimab combination treatment. Duration of stable disease for this patient was 4.2 months.

### Pharmacokinetics, immunogenicity, and pharmacodynamics

Fianlimab concentrations in the serum were similar when used alone or in combination with cemiplimab at 3 mg/kg Q3W or 350 mg Q3W (Supplementary Table S8; Supplementary Fig. S3). The pharmacokinetics of fianlimab were dose proportional over the dose range studied (1–40 mg/kg Q3W; Supplementary Fig. S4). Concentrations of cemiplimab in serum when administered in combination with fianlimab were similar to those observed with cemiplimab monotherapy in other studies (12, 13).

Immunogenicity rates against fianlimab were low (1.6%), with one patient developing treatment-emergent ADAs (indeterminate) at fianlimab 3 mg/kg Q3W of low titer (<1,000).

**Figure 3.**
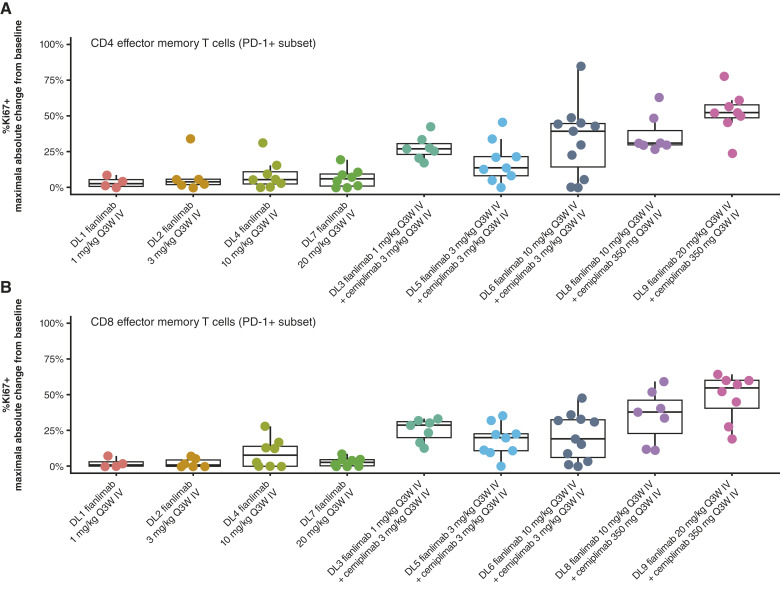
Analysis of T-cell subset proliferation subsequent to initiation of fianlimab as monotherapy or in combination with cemiplimab in (**A**) CD4 effector memory T cells and (**B**) CD8 effector memory T cells. Representative dot plots of CD4 and CD8^+^ T-cell memory subpopulations expressing Ki67+, LAG3+, HLA-DR+, PD-1+, or PD-L1+. Pharmacodynamic assay data from the 40 mg/kg dose cohort could not be generated due to poor quality of samples. DL, dose level; IV, intravenous; Q3W, every 3 weeks.

No pharmacodynamic effect on peripheral T cells was observed with fianlimab monotherapy. However, preliminary data suggest a dose-dependent relationship between fianlimab-plus-cemiplimab combination treatment and the proliferation of circulating PD-1–expressing CD4 and CD8 effector-memory (CCR7−) and central-memory (CCR7+) T-cell subsets (Fig. 3; Supplementary Fig. S5). There was a numerically but not statistically higher median value of proliferating T cells for the cohort treated with the highest dose of fianlimab (20 mg/kg compared with 10 mg/kg). No significant posttreatment changes were observed in regulatory T cells (CD4^+^/CD25+/FoxP3+) and naïve T cells (CD45RA+/CCR7+). No correlative trends were observed between clinical response and immunohistochemistry expression scores of LAG-3, PD-1, or MHC class II.

Based on safety, pharmacokinetic, and pharmacodynamic parameters, fianlimab 20 mg/kg or 1,600 mg fixed-dose equivalent Q3W was selected as the recommended phase 2 dose as monotherapy and in combination with cemiplimab 350 mg Q3W.

## Discussion

In this first-in-human phase 1 dose-escalation study, fianlimab-plus-cemiplimab combination therapy demonstrated an acceptable safety profile in patients with advanced malignancies (8). There were no unexpected safety signals with fianlimab monotherapy or fianlimab-plus-cemiplimab combination therapy compared with previous reports for cemiplimab (9). Preliminary clinical activity results were consistent with preclinical data, and support further research of the combination of anti-LAG-3 and anti-PD-1 therapy in patients with solid tumors.

The overall occurrence of any-grade TEAEs was generally similar between the monotherapy and combination therapy groups, with 90% (*n* = 20) of patients in the monotherapy group and 87% (*n* = 41) of patients in the combination therapy group experiencing a TEAE. The percentage of patients who received combination therapy and experienced a TEAE of hypothyroidism was greater than that generally seen with cemiplimab but within the range reported for other anti-PD-1 combination therapies (9, 11, 14, 15). The most frequent TRAEs associated with fianlimab were low-grade nausea and infusion-related reaction. Notably, in the combined first-in-human study population of 47 patients who received fianlimab plus cemiplimab in the dose-escalation cohorts, infusion-related reactions of any grade were observed in 15% of patients—within the range of previous reports for other immune checkpoint inhibitor therapies such as relatlimab plus anti-PD-1 (6%) and ipilimumab plus anti-PD-1 (5%–12%; refs. 11, 16). None of these events of infusion-related reactions led to permanent study drug discontinuation. One (2%) patient receiving combination therapy experienced a DLT; TRAEs of any grade occurred in 32 (68%) patients.

Although cross-trial comparisons should be made with caution, the safety profile of fianlimab plus cemiplimab was generally favorable relative to that of other immune checkpoint inhibitor combinations, with 68% (*n* = 32) of patients experiencing a TRAE of any grade and 11% (*n* = 5) of patients experiencing a grade ≥3 TRAE (Supplementary Table S7). In patients with advanced melanoma receiving relatlimab plus nivolumab, higher any-grade and grade ≥3 TRAE rates were observed (11). Ipilimumab (an anti-CTLA4 agent) plus nivolumab also demonstrated a less favorable safety profile in patients with advanced melanoma than fianlimab plus cemiplimab in the present study (17).

Fianlimab concentrations in serum were dose proportional over a dose range of 1 to 40 mg/kg Q3W and were similar in monotherapy and in combination with cemiplimab, indicating the absence of drug–drug interactions. The immunogenicity rate was low (1.6%), with one of 63 patients showing treatment-emergent ADAs. Of note, this study is the first in which such high doses of a LAG-3 inhibitor were tested and tolerated. Data suggest higher doses of anti-LAG-3, such as fianlimab, may be required for full-target saturation (18, 19).

Fianlimab monotherapy showed no pharmacodynamic effect on peripheral T cells. The combination therapy suggested a pharmacodynamic effect on activated peripheral T cells and a dose-dependent relationship between combination dosing with increasing doses of anti-LAG-3 and proliferating Ki-67+ circulating PD-1–expressing CD4 and CD8 effector memory T-cell subsets. It has been shown in published studies that anti-PD-1 monotherapy mediates proliferation in circulating PD-1+ T cells (Ki-67+; ref. 20). Therefore, the pharmacodynamic effect observed in this study is likely mediated by both antibodies. No correlative trends were observed between clinical response and immunohistochemistry expression scores of LAG-3, PD-1, or MHC class II, likely due to the small number of patients, low objective response rates, multiple tumor types, and different dose levels tested.

The preliminary antitumor results showed no responses with fianlimab monotherapy, which is consistent with previous reports of minimal, if any, activity for other LAG-3 inhibitors administered alone (21, 22). Limited clinical activity was also observed among patients treated with the fianlimab-plus-cemiplimab combination and likely reflects the patient population enrolled. Two (4%) patients in the fianlimab-plus-cemiplimab group had a partial response, and three (19%) patients who experienced disease progression with fianlimab monotherapy had a partial response after receiving fianlimab-plus-cemiplimab combination treatment.

Limitations of the dose-escalation part of this phase 1 study are: 60% of patients had received ≥3 lines of systemic therapy before enrollment; there were a variety of tumor types; and most patients had tumors that were not expected to have strong responses to anti-PD-1 monotherapy, including colorectal, pancreatic, ovarian, prostate, and breast cancers (23–27). Several questions were not addressed within the population studied in this phase 1 trial, such as responsiveness to single-agent anti-LAG-3 or the combination in treatment-naïve patients, and certain tumor types with high LAG-3 expression; the impact of high LAG-3 or PD-L1 expression also remains unclear (11, 21).

Following the acceptable safety data of this dose-escalation study, the cohort-expansion portion of the trial enrolled patients with select solid tumors and non–Hodgkin lymphoma with the aim of assessing clinical activity at the recommended phase 2 dose of fianlimab (1,600 mg) plus cemiplimab (350 mg) in both anti-PD-1/PD-L1-naïve and anti-PD-1/PD-L1-pretreated clinical settings.

In three ongoing expansion cohorts of patients with advanced melanoma, preliminary results from anti-PD-1/PD-L1-naïve patients reported an ORR of 61.2%, and patients who had disease progression after prior anti-PD-1 adjuvant treatment had an ORR of 61.5% (28). Also, as previously reported, the fianlimab-plus-cemiplimab combination showed an ORR of 13% in patients who received prior anti-PD-1/PD-L1 in the unresectable or metastatic setting (29).

This study met its prespecified objectives. These data support the potential for benefit from this combination, with less toxicity than anti-CTLA4-plus-anti-PD-1 combinations, and will be further explored in a phase 3 trial of fianlimab plus cemiplimab in patients with previously untreated locally advanced or metastatic melanoma (ClinicalTrials.gov identifier NCT05352672), a phase 3 trial of fianlimab plus cemiplimab as adjuvant therapy in patients with previously untreated unresectable advanced melanoma (ClinicalTrials.gov identifier NCT05608291), and a phase 3 trial of fianlimab plus cemiplimab in patients with advanced or metastatic melanoma (NCT06246916).

The results of this first-in-human dose-escalation study demonstrated an acceptable safety profile of fianlimab both as monotherapy and in combination with cemiplimab in patients with advanced malignancies. Fianlimab was tolerated by patients, and preliminary clinical activity results support findings from previous preclinical and clinical studies to provide evidence of a potential alternative anti-LAG-3 and anti-PD-1 combination therapy.

## Supplementary Material

Supplementary Material 1 Supplementary Material
