# Supplementary material for: First-in-Human Dose-Escalation Study of Fianlimab, an Antilymphocyte Activation Gene-3 Antibody, with Cemiplimab in Patients with Advanced Malignancies
Source: Clin Cancer Res. 2024 Oct 18;30(24):5601–11. doi: 10.1158/1078-0432.CCR-23-3883 (PMC11647203; doi:10.1158/1078-0432.CCR-23-3883)
Supplement: Supplementary Material 1 — Supplementary Material [file ccr-23-3883_supplementary_material_1_suppms1.docx]

# Supplementary materials

## Table of contents

| Supplementary Fig. S1. Study design |
| --- |
| Supplementary Fig. S2.  (A) Clinical activity and (B) changes to target lesion over time in patients treated with fianlimab monotherapy |
| Supplementary Fig. S3. Mean (standard deviation) concentration of fianlimab over time as monotherapy or in combination with cemiplimab (log scale) |
| Supplementary Fig. S4. Linear regression of mean concentration of functional fianlimab at C_trough_ in cycle 4 versus dose after IV administration as monotherapy or in combination with cemiplimab |
| Supplementary Fig. S5. Gating strategy for analysis of T cell subset proliferation |
| Supplementary Table S1. Baseline tumor characteristics |
| Supplementary Table S2. Patient disposition |
| Supplementary Table S3. Summary of treatment exposure |
| Supplementary Table S4. Treatment-emergent adverse events regardless of attribution |
| Supplementary Table S5. Treatment-emergent adverse events by dose level |
| Supplementary Table S6. Sponsor-identified immune-mediated adverse events |
| Supplementary Table S7. Treatment-related adverse events |
| Supplementary Table S8. Pharmacokinetic parameters for functional fianlimab concentration over time with monotherapy and combination treatment |
| Supplementary Table S9: Representativeness of study participants |

Supplementary Fig. S1. Study design


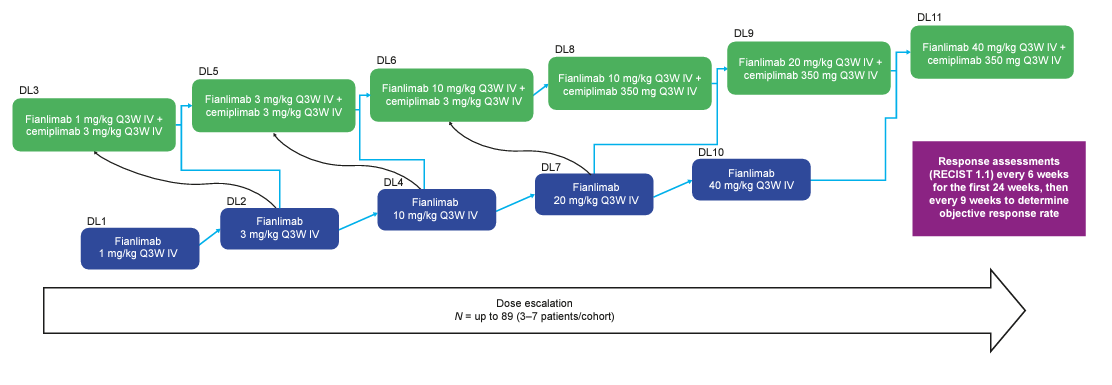


Abbreviations: DL, dose level; IV, intravenous; Q3W, every 3 weeks; RECIST 1.1, Response Evaluation Criteria in Solid Tumors version 1.1.

Supplementary Fig. S2. (A) Clinical activity and (B) changes to target lesion over time in patients treated with fianlimab monotherapy


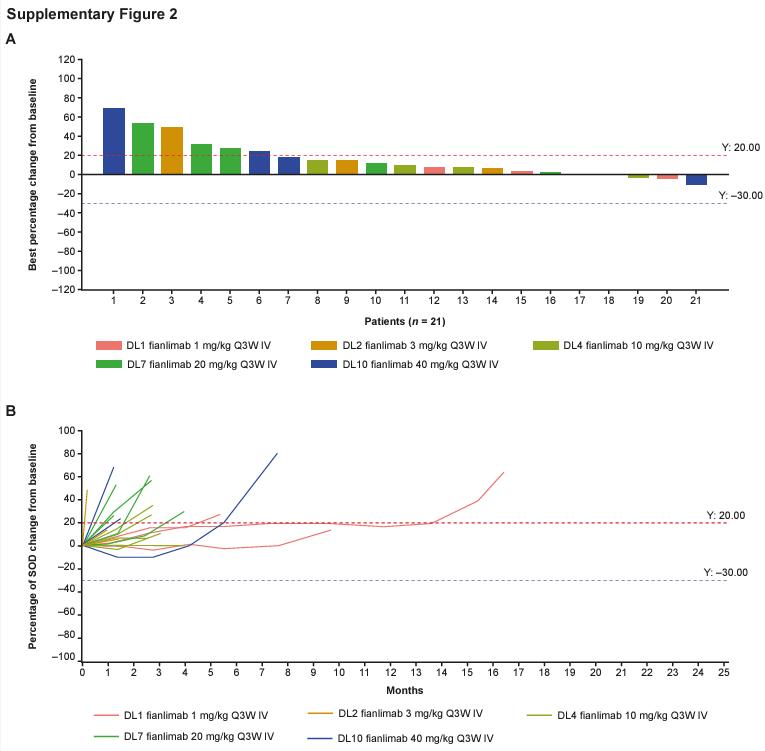


Figures only include patients who had both baseline and post-baseline target lesion assessments; not all patients had these assessments, therefore, some patients may not be shown in this figure. Eight patients (8/31; 25.8%) were not evaluated in this treatment group.

Abbreviations: DL, dose level; IV, intravenous; Q3W, every 3 weeks; SOD, sum of diameters.

Supplementary Fig. S3. Mean (standard deviation) concentration of fianlimab over time as monotherapy or in combination with cemiplimab (log scale)


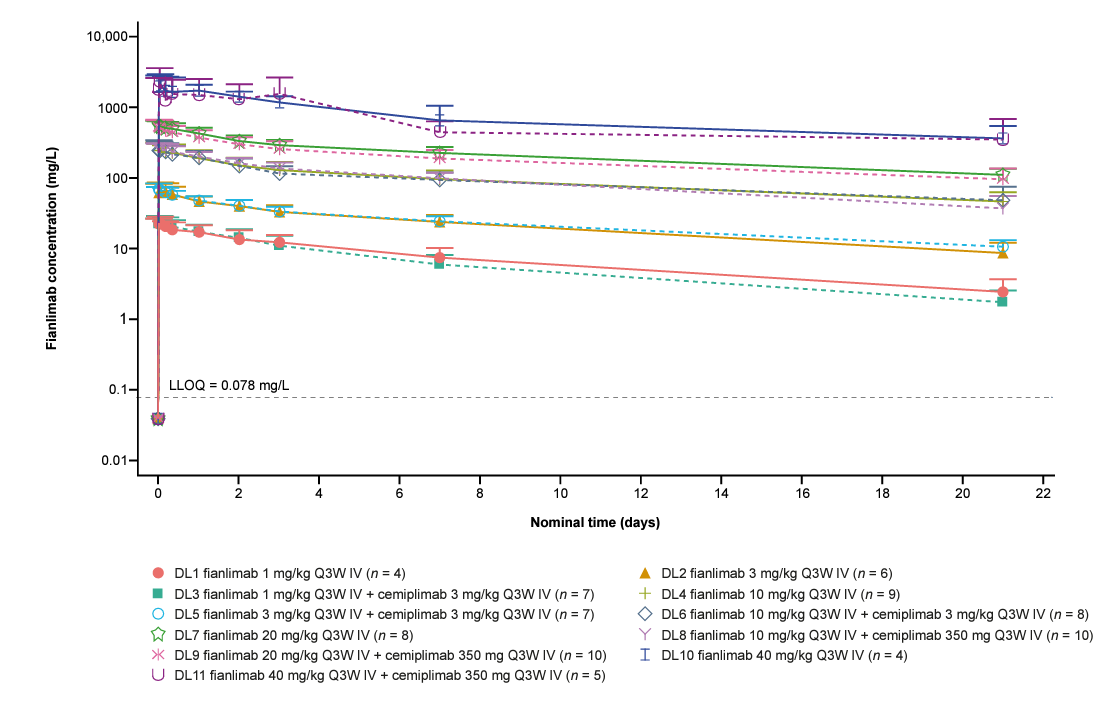


Concentrations below the LLOQ were set to LLOQ/2. Solid lines correspond to fianlimab monotherapy data. Dashed lines correspond to fianlimab plus cemiplimab combination data.

Abbreviations: DL, dose level; IV, intravenous; LLOQ, lower limit of quantitation; Q3W, every 3 weeks.

Supplementary Fig. S4. Linear regression of mean concentration of fianlimab at C_trough_ in cycle 4 versus dose after IV administration as monotherapy or in combination with cemiplimab


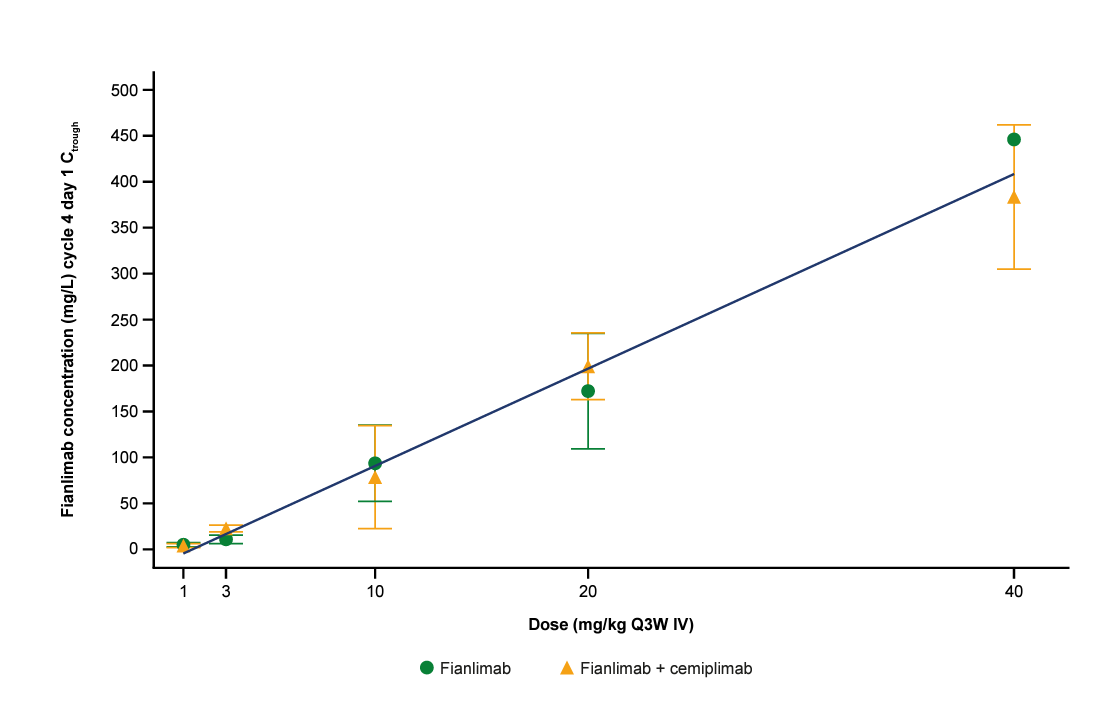


At cycle 4 day 1, C_trough_ is the pre-infusion concentration. The last pharmacokinetics collection date was August 25, 2021. R^2^ for mean peak concentration versus dose = 0.98687.

Abbreviations: C_trough_, trough concentration; IV, intravenous; Q3W, every 3 weeks.

Supplementary Fig. S5. Gating strategy for analysis of T cell subset proliferation


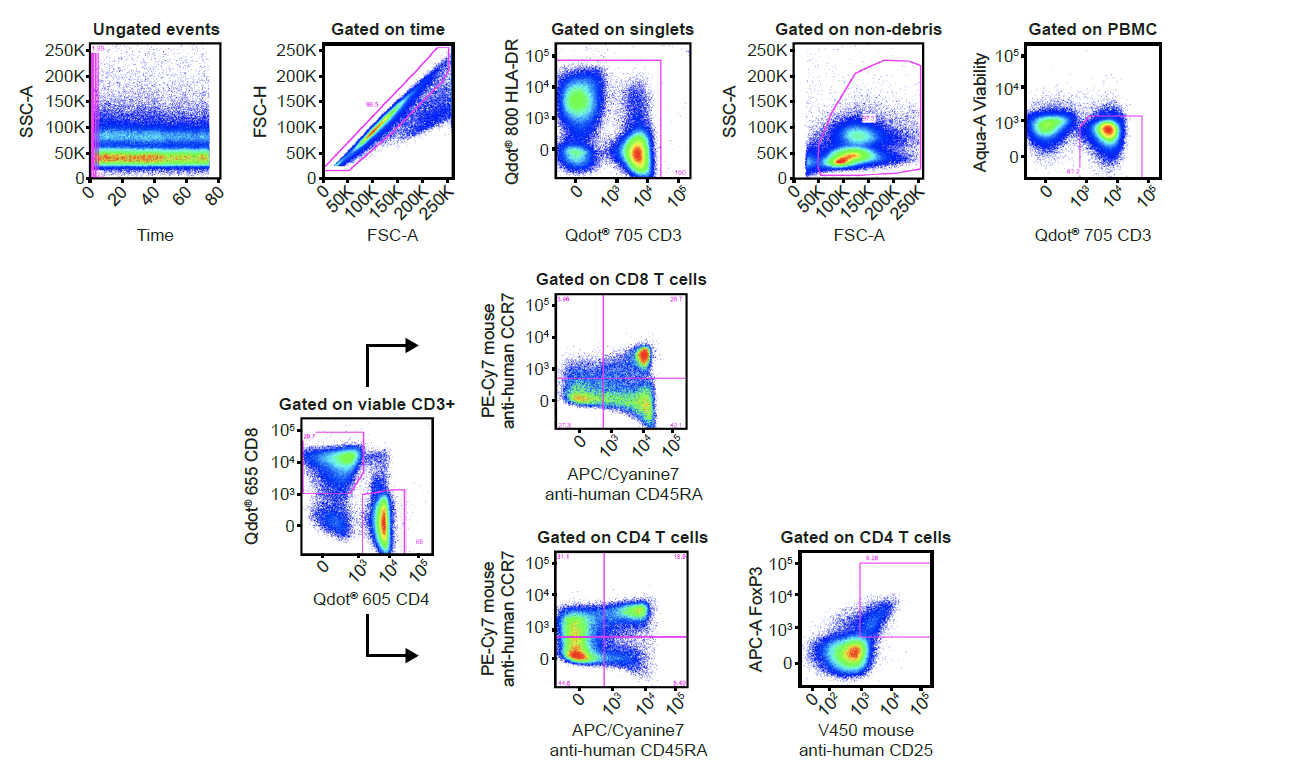


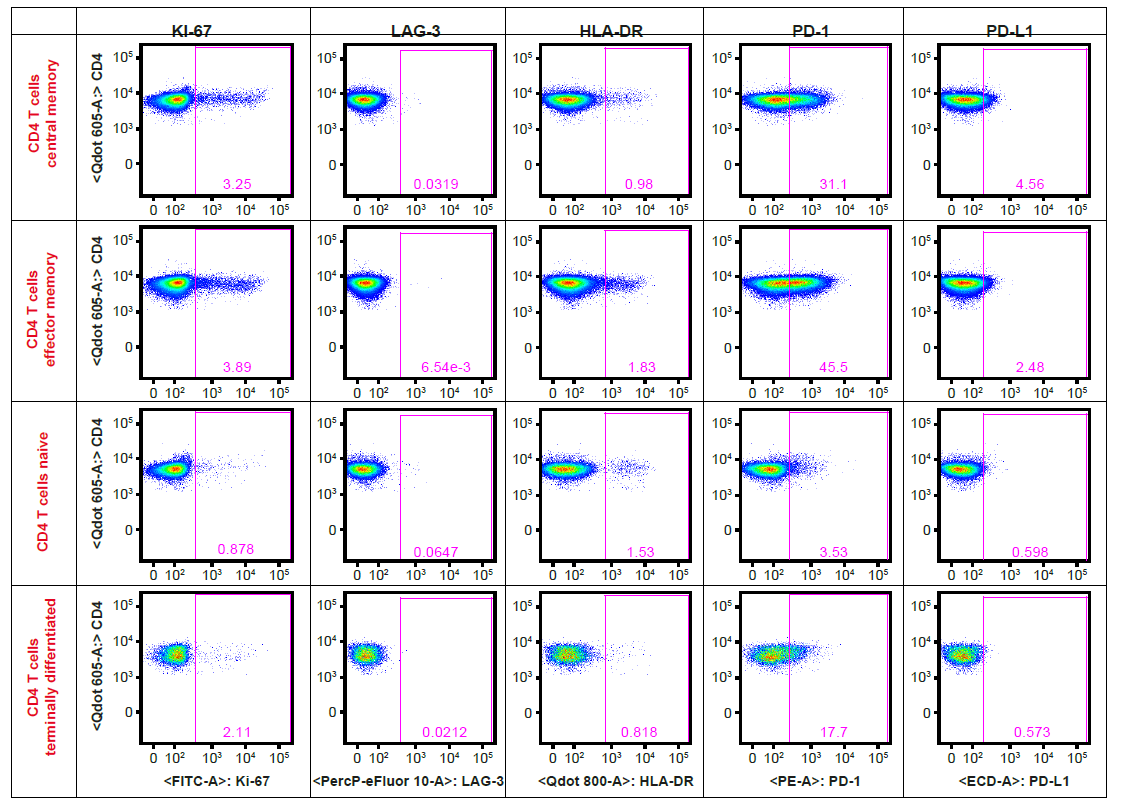


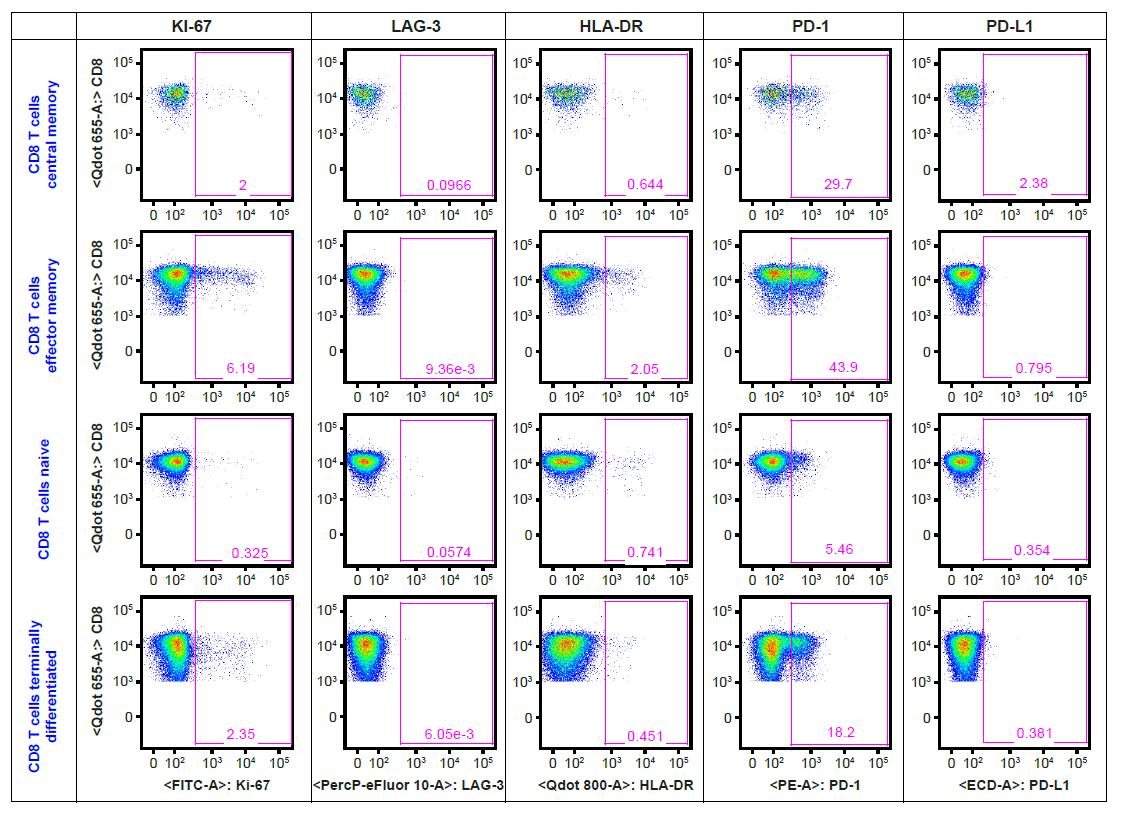


Gating strategy to assess in vivo activation of T cell memory subpopulations expressing Ki67+, LAG3+, HLA-DR +, PD-1+ or PD-L1+ in blood.

Supplementary Table S1. Baseline tumor characteristics

| Primary site of cancer, *n* (%) | Fianlimab monotherapy (*n* = 31) | Fianlimab + cemiplimab (*n* = 47) | Monotherapy to combination^a^ (*n* = 16) |
| --- | --- | --- | --- |
| Anus | 0 | 1 (2.1) | 0 |
| Appendix | 1 (3.2) | 0 | 0 |
| Biliary tract | 2 (6.5) | 3 (6.4) | 1 (6.3) |
| Bone | 1 (3.2) | 0 | 0 |
| Breast | 2 (6.5) | 2 (4.3) | 0 |
| Cervix | 0 | 2 (4.3) | 0 |
| Colon | 3 (9.7) | 11 (23.4) | 3 (18.8) |
| Esophagus | 0 | 2 (4.3) | 0 |
| Head and neck | 0 | 1 (2.1) | 0 |
| Liver | 2 (6.5) | 1 (2.1) | 2 (12.5) |
| Lung | 3 (9.7) | 4 (8.5) | 2 (12.5) |
| Other | 3 (9.7) | 2 (4.3) | 2 (12.5) |
| Ovary | 2 (6.5) | 2 (4.3) | 1 (6.3) |
| Pancreas | 1 (3.2) | 3 (6.4) | 0 |
| Prostate | 2 (6.5) | 1 (2.1) | 0 |
| Rectum | 0 | 3 (6.4) | 0 |
| Retroperitoneum | 1 (3.2) | 1 (2.1) | 0 |
| Salivary gland | 0 | 2 (4.3) | 0 |
| Skin melanoma | 0 | 1 (2.1) | 0 |
| Skin nonmelanoma | 1 (3.2) | 1 (2.1) | 1 (6.3) |
| Stomach | 1 (3.2) | 0 | 0 |
| Thyroid | 0 | 2 (4.3) | 0 |
| Uterus | 3 (9.7) | 1 (2.1) | 2 (12.5) |
| Vagina | 1 (3.2) | 1 (2.1) | 1 (6.3) |

^a^A subset of patients who received fianlimab monotherapy as primary treatment, then subsequently received fianlimab plus cemiplimab combination therapy during a re-treatment phase. Data presented were gathered from the period during which patients were receiving combination therapy.

Supplementary Table S2. Patient disposition

| *n* (%) | Fianlimab monotherapy (*n* = 31) | Fianlimab + cemiplimab (*n* = 47) | Monotherapy to combination^a^ (*n* = 16) |
| --- | --- | --- | --- |
| Completed treatment | 1 (3.2) | 0 | 0 |
| Discontinued treatment | 30 (96.8) | 47 (100.0) | 16 (100.0) |
| Primary reason for discontinuing treatment | | | |
| Adverse event | 0 | 1 (2.1) | 2 (12.5) |
| Physician decision | 2 (6.5) | 1 (2.1) | 1 (6.3) |
| Disease progression | 20 (64.5) | 38 (80.9) | 10 (62.5) |
| Death | 4 (12.9) | 3 (6.4) | 0 |
| Other | 2 (6.5) | 0 | 1 (6.3) |
| Withdrawal of consent | 2 (6.5) | 3 (6.4) | 0 |
| Patient decision | 0 | 1 (2.1) | 2 (12.5) |

^a^A subset of patients who received fianlimab monotherapy as primary treatment, then subsequently received fianlimab plus cemiplimab combination therapy during a re-treatment phase. Data presented were gathered from the period during which patients were receiving combination therapy.

Supplementary Table S3. Summary of treatment exposure

| *n* (%) | Fianlimab monotherapy (*n* = 31) | Fianlimab + cemiplimab (*n* = 47) | Monotherapy to combination^a^ (*n* = 16) |
| --- | --- | --- | --- |
| Duration of exposure, weeks |  |  |  |
| Median (range) | 11.3 (2–51) | 9.0 (3–95) | 15.1 (3–66) |
| IQR | 6.0–15.9 | 6.0–18.0 | 6.3–30.0 |
| Number of doses of study drug, median (range) | 4 (1–17) | 3 (1–25) | 5 (1–20) |

^a^A subset of patients who received fianlimab monotherapy as primary treatment, then subsequently received fianlimab + cemiplimab combination therapy during a re-treatment phase. Data presented were gathered from the period of time during which patients were receiving combination therapy.

Abbreviation: IQR, interquartile range.

Supplementary Table S4. Treatment-emergent adverse events regardless of attribution

| ***n* (%)** | **Fianlimab monotherapy (*n* = 31)** | | **Fianlimab + cemiplimab (*n* = 47)** | | **Monotherapy to combination^a^ (*n* = 16)** | |
| --- | --- | --- | --- | --- | --- | --- |
|  | **Any grade** | **Grade ≥3** | **Any grade** | **Grade ≥3** | **Any grade** | **Grade ≥3** |
| Any | 28 (90.3) | 12 (38.7) | 41 (87.2) | 22 (46.8) | 14 (87.5) | 6 (37.5) |
| Serious | 6 (19.4) | 5 (16.1) | 7 (14.9) | 7 (14.9) | 3 (18.8) | 3 (18.8) |
| Led to discontinuation | 0 | 0 | 0 | 0 | 2 (12.5) | N/A |
| With an outcome of death | 1 (3.2) | 1 (3.2) | 1 (2.1) | 1 (2.1) | 0 | 0 |
| Occurred in ≥5% of patients in any group ordered by frequency in patients who received fianlimab monotherapy | | | | | | |
| Nausea | 7 (22.6) | 1 (3.2) | 10 (21.3) | 1 (2.1) | 4 (25.0) | 1 (6.3) |
| Abdominal pain | 6 (19.4) | 0 | 2 (4.3) | 0 | 1 (6.3) | 0 |
| Decreased appetite | 6 (19.4) | 0 | 8 (17.0) | 2 (4.3) | 4 (25.0) | 1 (6.3) |
| Diarrhea | 5 (16.1) | 0 | 8 (17.0) | 0 | 4 (25.0) | 0 |
| Fatigue | 5 (16.1) | 0 | 17 (36.2) | 1 (2.1) | 7 (43.8) | 0 |
| Vomiting | 5 (16.1) | 1 (3.2) | 8 (17.0) | 1 (2.1) | 3 (18.8) | 0 |
| Cystitis | 5 (10.6) | 0 | 5 (10.6) | 0 | 1 (6.3) | 1 (6.3) |
| Anemia | 4 (12.9) | 3 (9.7) | 7 (14.9) | 3 (6.4) | 0 | 0 |
| Back pain | 4 (12.9) | 0 | 1 (2.1) | 0 | 0 | 0 |
| Dyspnea | 4 (12.9) | 1 (3.2) | 3 (6.4) | 2 (4.3) | 0 | 0 |
| Nasal congestion | 4 (12.9) | 0 | 1 (2.1) | 0 | 1 (6.3) | 0 |
| Blood alkaline phosphatase increased | 3 (9.7) | 2 (6.5) | 2 (4.3) | 1 (2.1) | 0 | 0 |
| Constipation | 3 (9.7) | 0 | 6 (12.8) | 0 | 0 | 0 |
| Cough | 3 (9.7) | 0 | 5 (10.6) | 1 (2.1) | 2 (12.5) | 0 |
| Headache | 3 (9.7) | 0 | 7 (14.9) | 0 | 2 (12.5) | 0 |
| Hypertension | 3 (9.7) | 2 (6.5) | 0 | 0 | 1 (6.3) | 0 |
| Hypotension | 3 (9.7) | 0 | 2 (4.3) | 1 (2.1) | 2 (12.5) | 0 |
| Infusion-related reaction | 3 (9.7) | 0 | 7 (14.9) | 0 | 2 (12.5) | 0 |
| Lymphocyte count decreased | 3 (9.7) | 3 (9.7) | 4 (8.5) | 2 (4.3) | 0 | 0 |
| Neck pain | 3 (9.7) | 0 | 0 | 0 | 0 | 0 |
| Abdominal distension | 2 (6.5) | 0 | 1 (2.1) | 0 | 2 (12.5) | 0 |
| Abdominal pain upper | 2 (6.5) | 1 (3.2) | 1 (2.1) | 0 | 0 | 0 |
| Blood creatinine increased | 2 (6.5) | 1 (3.2) | 3 (6.4) | 0 | 0 | 0 |
| Bronchitis | 2 (6.5) | 1 (3.2) | 0 | 0 | 1 (6.3) | 0 |
| Dehydration | 2 (6.5) | 0 | 2 (4.3) | 2 (4.3) | 2 (12.5) | 0 |
| Depression | 2 (6.5) | 0 | 1 (2.1) | 0 | 0 | 0 |
| Hypokalemia | 2 (6.5) | 0 | 5 (10.6) | 1 (2.1) | 2 (12.5) | 2 (12.5) |
| Hypomagnesemia | 2 (6.5) | 0 | 3 (6.4) | 0 | 1 (6.3) | 0 |
| Hyponatremia | 2 (6.5) | 1 (3.2) | 5 (10.6) | 3 (6.4) | 1 (6.3) | 1 (6.3) |
| Insomnia | 2 (6.5) | 0 | 4 (8.5) | 0 | 0 | 0 |
| Night sweats | 2 (6.5) | 1 (3.2) | 0 | 0 | 0 | 0 |
| Edema peripheral | 2 (6.5) | 0 | 5 (10.6) | 0 | 4 (25.0) | 0 |
| Oropharyngeal pain | 2 (6.5) | 0 | 1 (2.1) | 0 | 1 (6.3) | 0 |
| Pruritus | 2 (6.5) | 0 | 2 (4.3) | 0 | 1 (6.3) | 0 |
| Tumor pain | 2 (6.5) | 0 | 5 (10.6) | 0 | 0 | 0 |
| Ascites | 1 (3.2) | 0 | 2 (4.3) | 2 (4.3) | 1 (6.3) | 0 |
| Aspartate aminotransferase increased | 1 (3.2) | 1 (3.2) | 3 (6.4) | 0 | 0 | 0 |
| Chills | 1 (3.2) | 0 | 7 (14.9) | 0 | 0 | 0 |
| Flank pain | 1 (3.2) | 0 | 2 (4.3) | 0 | 1 (6.3) | 0 |
| Hyperglycemia | 1 (3.2) | 1 (3.2) | 3 (6.4) | 2 (4.3) | 1 (6.3) | 1 (6.3) |
| Myalgia | 1 (3.2) | 0 | 3 (6.4) | 0 | 0 | 0 |
| Pain in extremity | 1 (3.2) | 0 | 3 (6.4) | 1 (2.1) | 0 | 0 |
| Pneumonia | 1 (3.2) | 1 (3.2) | 3 (6.4) | 0 | 0 | 0 |
| Pulmonary embolism | 1 (3.2) | 1 (3.2) | 2 (4.3) | 1 (2.1) | 1 (6.3) | 0 |
| Pyrexia | 1 (3.2) | 0 | 6 (12.8) | 0 | 0 | 0 |
| Rash maculo-papular | 1 (3.2) | 0 | 1 (2.1) | 0 | 5 (31.3) | 2 (12.5) |
| Urinary tract infection | 1 (3.2) | 0 | 6 (12.8) | 3 (6.4) | 0 | 0 |
| Adrenal insufficiency | 0 | 0 | 2 (4.3) | 0 | 1 (6.3) | 1 (6.3) |
| Aphthous ulcer | 0 | 0 | 0 | 0 | 1 (6.3) | 1 (6.3) |
| Arthralgia | 0 | 0 | 3 (6.4) | 0 | 3 (18.8) | 0 |
| Asthenia | 0 | 0 | 4 (8.5) | 1 (2.1) | 0 | 0 |
| COVID-19 | 0 | 0 | 0 | 0 | 1 (6.3) | 0 |
| Decubitus ulcer | 0 | 0 | 1 (2.1) | 0 | 1 (6.3) | 0 |
| Dermatitis contact | 0 | 0 | 1 (2.1) | 0 | 1 (6.3) | 0 |
| Dizziness | 0 | 0 | 4 (8.5) | 0 | 1 (6.3) | 0 |
| Dry mouth | 0 | 0 | 1 (2.1) | 0 | 1 (6.3) | 0 |
| Duodenitis | 0 | 0 | 0 | 0 | 1 (6.3) | 0 |
| Ear infection | 0 | 0 | 0 | 0 | 1 (6.3) | 0 |
| Fall | 0 | 0 | 1 (2.1) | 0 | 2 (12.5) | 0 |
| Hematoma | 0 | 0 | 0 | 0 | 1 (6.3) | 0 |
| Hyperhidrosis | 0 | 0 | 3 (6.4) | 0 | 1 (6.3) | 0 |
| Hypothyroidism | 0 | 0 | 7 (14.9) | 1 (2.1) | 1 (6.3) | 0 |
| Muscle spasms | 0 | 0 | 0 | 0 | 1 (6.3) | 0 |
| Nail infection | 0 | 0 | 0 | 0 | 1 (6.3) | 0 |
| Esophageal ulcer | 0 | 0 | 0 | 0 | 1 (6.3) | 0 |
| Pneumonitis | 0 | 0 | 0 | 0 | 1 (6.3) | 1 (6.3) |
| Rotator cuff syndrome | 0 | 0 | 0 | 0 | 1 (6.3) | 0 |
| Septic shock | 0 | 0 | 0 | 0 | 1 (6.3) | 1 (6.3) |
| Sinusitis | 0 | 0 | 1 (2.1) | 0 | 1 (6.3) | 0 |
| Skin infection | 0 | 0 | 1 (2.1) | 0 | 2 (12.5) | 0 |
| Subcutaneous abscess | 0 | 0 | 0 | 0 | 1 (6.3) | 0 |
| Thrombophlebitis superficial | 0 | 0 | 0 | 0 | 1 (6.3) | 0 |
| Transaminases increased | 0 | 0 | 0 | 0 | 1 (6.3) | 0 |
| Upper respiratory tract infection | 0 | 0 | 1 (2.1) | 0 | 1 (6.3) | 0 |
| Urethral atrophy | 0 | 0 | 0 | 0 | 1 (6.3) | 0 |
| Vertigo positional | 0 | 0 | 0 | 0 | 1 (6.3) | 0 |
| Weight increased | 0 | 0 | 0 | 0 | 1 (6.3) | 0 |

^a^A subset of patients who received fianlimab monotherapy as primary treatment, then subsequently received fianlimab plus cemiplimab combination therapy during a re-treatment phase. Data presented were gathered from the period during which patients were receiving combination therapy.

Abbreviations: IV, intravenous; N/A, not available; Q3W, every 3 weeks.

Supplementary Table S5. Treatment-emergent adverse events by treatment dose level

| **Fianlimab monotherapy** | | | | | | | | | | | | |
| --- | --- | --- | --- | --- | --- | --- | --- | --- | --- | --- | --- | --- |
| Patients with any TEAE, *n* (%) | **DL1 fianlimab 1 mg/kg Q3W IV (*n* = 4)** | | **DL2 fianlimab 3 mg/kg Q3W IV (*n* = 6)** | | **DL4 fianlimab 10 mg/kg Q3W IV (*n* = 9)** | |  | | **DL7 fianlimab 20 mg/kg Q3W IV  (*n* = 8)** | | **DL10 fianlimab 40 mg/kg Q3W IV  (*n* = 4)** | |
|  | **All grades** | **Grade ≥3** | **All grades** | **Grade ≥3** | **All grades** | **Grade ≥3** |  |  | **All grades** | **Grade ≥3** | **All grades** | **Grade ≥3** |
|  | 4 (100.0) | 1 (25.0) | 5 (83.3) | 4 (66.7) | 8 (88.9) | 3 (33.3) |  |  | 7 (87.5) | 4 (50.0) | 4 (100.0) | 0 |
| **Fianlimab + cemiplimab combination therapy** | | | | | | | | | | | | |
| Patients with any TEAE, *n*(%) | **DL3 fianlimab 1 mg/kg Q3W IV + cemiplimab 3 mg/kg  (*n* = 7)** | | **DL5 fianlimab 3 mg/kg Q3W IV + cemiplimab 3 mg/kg  (*n* = 7)** | | **DL6 fianlimab 10 mg/kg Q3W IV + cemiplimab 3 mg/kg  (*n* = 8)** | | **DL8 fianlimab 10 mg/kg Q3W IV + cemiplimab 350 mg  (*n* = 10)** | | **DL9 fianlimab 20 mg/kg Q3W IV + cemiplimab 350 mg  (*n* = 10)** | | **DL11 fianlimab 40 mg/kg Q3W IV + cemiplimab 350 mg  (*n* = 5)** | |
|  | **All grades** | **Grade ≥3** | **All grades** | **Grade ≥3** | **All grades** | **Grade ≥3** | **All grades** | **Grade ≥3** | **All grades** | **Grade ≥3** | **All grades** | **Grade ≥3** |
|  | 5 (71.4) | 5 (71.4) | 6 (85.7) | 3 (42.9) | 8 (100.0) | 5 (62.5) | 8 (80.0) | 3 (30.0) | 10 (100.0) | 5 (50.0) | 4 (80.0) | 1 (20.0) |
| Fianlimab monotherapy to fianlimab + cemiplimab combination therapy^a^ | | | | | | | | | | | | |
| Patients with any TEAE, *n* (%) | **DL3 fianlimab 1 mg/kg Q3W IV + cemiplimab 3 mg/kg  (*n* = 2)** | | **DL5 fianlimab 3 mg/kg Q3W IV + cemiplimab 3 mg/kg  (*n* = 5)** | | **DL6 fianlimab 10 mg/kg Q3W IV + cemiplimab 3 mg/kg  (*n* = 6)** | |  | | **DL9 fianlimab 20 mg/kg Q3W IV + cemiplimab 350 mg  (*n* = 2)** | | **DL11 fianlimab 40 mg/kg Q3W IV + cemiplimab 350 mg  (*n* = 1)** | |
|  | **All grades** | **Grade ≥3** | **All grades** | **Grade ≥3** | **All grades** | **Grade ≥3** |  |  | **All grades** | **Grade ≥3** | **All grades** | **Grade ≥3** |
|  | 2 (100.0) | 0 | 5 (100.0) | 4 (80.0 | 4 (66.7) | 2 (33.3) |  |  | 2 (100.0) | 0 | 1 (100.0) | 0 |

^a^A subset of patients who received fianlimab monotherapy as primary treatment, then subsequently received fianlimab plus cemiplimab combination therapy during a re-treatment phase. Data presented were gathered from the period during which patients were receiving combination therapy.

Abbreviations: DL, dose level; IV, intravenous; Q3W, every 3 weeks; TEAE, treatment-emergent adverse event.

Supplementary Table S6. Sponsor-identified immune-mediated adverse events

| *n* (%) | Fianlimab monotherapy (*n* = 31) | | Fianlimab + cemiplimab (*n* = 47) | | Monotherapy to combination^a^ (*n* = 16) | |
| --- | --- | --- | --- | --- | --- | --- |
|  | **Any grade** | **Grade ≥3** | **Any grade** | **Grade ≥3** | **Any grade** | **Grade ≥3** |
| Any | 3 (9.7) | 1 (3.2) | 15 (31.9) | 3 (6.4) | 10 (62.5) | 3 (18.8) |
| Alanine aminotransferase increased | 1 (3.2) | 1 (3.2) | 0 | 0 | 0 | 0 |
| Adrenal insufficiency | 0 | 0 | 2 (4.3) | 0 | 1 (6.3) | 1 (6.3) |
| Arthralgia | 0 | 0 | 1 (2.1) | 0 | 3 (18.8) | 0 |
| Aspartate aminotransferase increased | 1 (3.2) | 1 (3.2) | 1 (2.1) | 0 | 0 | 0 |
| Blood alkaline phosphatase increased | 1 (3.2) | 0 | 0 | 0 | 0 | 0 |
| Diarrhea | 0 | 0 | 3 (6.4) | 0 | 4 (25.0) | 0 |
| Diabetic ketoacidosis | 0 | 0 | 1 (2.1) | 1 (2.1) | 0 | 0 |
| Hyperthyroidism | 0 | 0 | 1 (2.1) | 0 | 0 | 0 |
| Hypothyroidism | 0 | 0 | 7 (14.9) | 1 (2.1) | 1 (6.3) | 0 |
| Myasthenic syndrome | 0 | 0 | 1 (2.1) | 1 (2.1) | 0 | 0 |
| Myalgia | 0 | 0 | 2 (4.3) | 0 | 0 | 0 |
| Pneumonitis | 0 | 0 | 0 | 0 | 1 (6.3) | 1 (6.3) |
| Pruritis | 1 (3.2) | 0 | 0 | 0 | 1 (6.3) | 0 |
| Rash maculo-papular | 0 | 0 | 0 | 0 | 4 (25.0) | 2 (12.5) |
| Type 1 diabetes mellitus | 0 | 0 | 1 (2.1) | 1 (2.1) | 0 | 0 |

^a^A subset of patients who received fianlimab monotherapy as primary treatment, then subsequently received fianlimab plus cemiplimab combination therapy during a re-treatment phase. Data presented were gathered from the period during which patients were receiving combination therapy.

Supplementary Table S7. Treatment-related adverse events

| *n* (%) | Fianlimab  (*n* = 31) | | Fianlimab + cemiplimab  (*n* = 47) | | Monotherapy to combination^a^ (*n* = 16) | |
| --- | --- | --- | --- | --- | --- | --- |
|  | **Any grade** | **Grade ≥3** | **Any grade** | **Grade ≥3** | **Any grade** | **Grade ≥3** |
| Any | 11 (35.5) | 2 (6.5) | 32 (68.1) | 5 (10.6) | 11 (68.8) | 4 (25.0) |
| Serious | 0 | 0 | 3 (6.4) | 3 (6.4) | 2 (12.5) | 2 (12.5) |
| TRAEs ordered by frequency in patients who received fianlimab monotherapy | | | | | | |
| Infusion-related reaction | 3 (9.7) | 0 | 7 (14.9) | 0 | 1 (6.3) | 0 |
| Nausea | 3 (9.7) | 0 | 2 (4.3) | 0 | 1 (6.3) | 0 |
| Alanine aminotransferase increased | 1 (3.2) | 1 (3.2) | 0 | 0 | 0 | 0 |
| Anemia | 1 (3.2) | 1 (3.2) | 3 (6.4) | 1 (2.1) | 0 | 0 |
| Aspartate aminotransferase increased | 1 (3.2) | 1 (3.2) | 1 (2.1) | 0 | 0 | 0 |
| Blood alkaline phosphatase increased | 1 (3.2) | 0 | 0 | 0 | 0 | 0 |
| Fatigue | 1 (3.2) | 0 | 8 (17.0) | 0 | 6 (37.5) | 0 |
| Hyponatremia | 1 (3.2) | 0 | 0 | 0 | 0 | 0 |
| Leukocytosis | 1 (3.2) | 0 | 0 | 0 | 0 | 0 |
| Onycholysis | 1 (3.2) | 0 | 0 | 0 | 0 | 0 |
| Pruritus | 1 (3.2) | 0 | 0 | 0 | 1 (6.3) | 0 |
| Viral upper respiratory tract infection | 1 (3.2) | 0 | 0 | 0 | 0 | 0 |
| Vomiting | 1 (3.2) | 0 | 0 | 0 | 1 (6.3) | 0 |
| Adrenal insufficiency | 0 | 0 | 2 (4.3) | 0 | 1 (6.3) | 1 (6.3) |
| Aphthous ulcer | 0 | 0 | 0 | 0 | 1 (6.3) | 1 (6.3) |
| Arthralgia | 0 | 0 | 1 (2.1) | 0 | 3 (18.8) | 0 |
| Blood creatine phosphokinase increased | 0 | 0 | 1 (2.1) | 1 (2.1) | 0 | 0 |
| Chills | 0 | 0 | 1 (2.1) | 0 | 0 | 0 |
| Conjunctivitis | 0 | 0 | 1 (2.1) | 0 | 0 | 0 |
| Cystitis | 0 | 0 | 2 (4.3) | 0 | 0 | 0 |
| Decreased appetite | 0 | 0 | 3 (6.4) | 0 | 1 (6.3) | 0 |
| Diabetic ketoacidosis | 0 | 0 | 1 (2.1) | 1 (2.1) | 0 | 0 |
| Diarrhea | 0 | 0 | 3 (6.4) | 0 | 4 (25.0) | 0 |
| Dysgeusia | 0 | 0 | 1 (2.1) | 0 | 0 | 0 |
| Dyspepsia | 0 | 0 | 1 (2.1) | 0 | 0 | 0 |
| Dyspnea | 0 | 0 | 1 (2.1) | 0 | 0 | 0 |
| Headache | 0 | 0 | 1 (2.1) | 0 | 1 (6.3) | 0 |
| Hyperhidrosis | 0 | 0 | 1 (2.1) | 0 | 0 | 0 |
| Hyperthyroidism | 0 | 0 | 1 (2.1) | 0 | 0 | 0 |
| Hyponatremia | 0 | 0 | 0 | 0 | 0 | 0 |
| Hypothyroidism | 0 | 0 | 7 (14.9) | 1 (2.1) | 1 (6.3) | 0 |
| Lymphocyte count decreased | 0 | 0 | 2 (4.3) | 1 (2.1) | 0 | 0 |
| Malaise | 0 | 0 | 1 (2.1) | 0 | 0 | 0 |
| Myalgia | 0 | 0 | 2 (4.3) | 0 | 0 | 0 |
| Myasthenic syndrome | 0 | 0 | 1 (2.1) | 1 (2.1) | 0 | 0 |
| Nail infection | 0 | 0 | 0 | 0 | 1 (6.3) | 0 |
| Neutrophil count decreased | 0 | 0 | 1 (2.1) | 0 | 0 | 0 |
| Pneumonitis | 0 | 0 | 0 | 0 | 1 (6.3) | 1 (6.3) |
| Pyrexia | 0 | 0 | 1 (2.1) | 0 | 1 (6.3) | 0 |
| Rash maculo-papular | 0 | 0 | 0 | 0 | 4 (25.0) | 2 (12.5) |
| Skin infection | 0 | 0 | 0 | 0 | 1 (6.3) | 0 |
| Type I diabetes mellitus | 0 | 0 | 1 (2.1) | 1 (2.1) | 0 | 0 |

^a^A subset of patients who received fianlimab monotherapy as primary treatment, then subsequently received fianlimab plus cemiplimab combination therapy during a re-treatment phase. Data presented were gathered from the period during which patients were receiving combination therapy.

Abbreviations: DL, dose level; IV, intravenous; Q3W, every 3 weeks; TRAE, treatment-related adverse event.

Supplementary Table S8. Pharmacokinetic parameters of functional fianlimab concentration over time with monotherapy and combination treatment

| **Dose-escalation cohorts (Q3W dose)** | **After the first dose** | | | | **At cycle 4 day 1** | | | |
| --- | --- | --- | --- | --- | --- | --- | --- | --- |
|  | **C_trough_, mg/L** | | **C_max_, mg/L** | | **C_trough_, mg/L** | | **C_max_, mg/L** | |
|  | **n** | **Mean (SD)** | **n** | **Mean (SD)** | **n** | **Mean (SD)** | **n** | **Mean (SD)** |
| DL1 fianlimab 1 mg/kg | 4 | 2.4 (1.3) | 4 | 23.0 (5.2) | 4 | 5.0 (2.3) | 4 | 30.1 (6.2) |
| DL2 fianlimab 3 mg/kg | 4 | 8.6 (3.4) | 6 | 65.2 (21.8) | 2 | 10.9 (4.7) | 1 | 79.9 (---) |
| DL3 fianlimab 1 mg/kg + cemiplimab 3 mg/kg | 7 | 1.7 (0.8) | 7 | 23.4 (5.6) | 2 | 4.0 (2.2) | 2 | 32.0 (9.4) |
| DL4 fianlimab 10 mg/kg | 7 | 46.4 (16.3) | 9 | 251 (66.8) | 6 | 93.6 (41.5) | 6 | 360 (82.0) |
| DL5 fianlimab 3 mg/kg + cemiplimab 3 mg/kg | 6 | 10.6 (2.5) | 7 | 72.5 (11.9) | 3 | 22.7 (3.7) | 3 | 109 (19.7) |
| DL6 fianlimab 10 mg/kg + cemiplimab 3 mg/kg | 7 | 48.2 (26.6) | 8 | 255 (91.6) | 3 | 93.1 (57.7) | 2 | 353 (103) |
| DL7 fianlimab 20 mg/kg | 7 | 110 (24.1) | 8 | 563 (101) | 4 | 172 (62.7) | 4 | 701 (195) |
| DL8 fianlimab 10 mg/kg + cemiplimab 350 mg | 9 | 37.0 (18.3) | 10 | 269 (53.6) | 3 | 64.3 (62.4) | 3 | 273 (103) |
| DL9 fianlimab 20 mg/kg + cemiplimab 350 mg | 10 | 95.7 (40.2) | 10 | 539 (137) | 6 | 199 (36.3) | 6 | 675 (139) |
| DL10 fianlimab 40 mg/kg | 4 | 362 (179) | 4 | 2165 (651) | 1 | 446 (---) | 1 | 1470 (---) |
| DL11 fianlimab 40 mg/kg + cemiplimab 350 mg | 3 | 349 (335) | 5 | 2552 (1374) | 2 | 384 (78.5) | 2 | 1345 (290) |

Below the lower limit of quantitation was set to 0. Last pharmacokinetics collection date: August 25, 2021. C_max_ after the first dose is at cycle 1, C_trough_ after the first dose is at cycle 2 day 1 pre-infusion, C_max_ at cycle 4 day 1 is at the end of infusion, and C_trough_ at cycle 4 day 1 is the pre-infusion concentration.

Abbreviations: C_max_, maximum concentration; C_trough_, trough concentration; DL, dose level; Q3W, every 3 weeks; SD, standard deviation.

Supplementary Table S9. Representativeness of study participants

| **Cancer type(s)/subtype(s)/ stage(s)/condition(s) related to** | **Advanced solid tumors and lymphoma** |
| --- | --- |
| Sex | Globally, 10.1 million new cancer cases were reported in males in 2020, with the most commonly diagnosed cancer types being lung, prostate, and colorectal cancer. In females, about 9.2 million new cancer cases were detected, with breast, colorectal, and lung cancer having the highest incidence^1^ |
| Age | The median age of cancer diagnosis is 66 years for all tumor types in the USA^2^ |
| Race/ethnicity | In the USA, in all ethnic groups, African American males and white females present the most new cancer cases and Asian/Pacific Islander (of both sexes) the least |
| Geography | Cancer is the second leading cause of death in the USA and worldwide |
| Other considerations | Immune checkpoint inhibitor treatments have limited response rates, and treatment resistance over time is commonly observed. New combination treatments targeting different immune checkpoint pathways in one treatment regimen are being trialed to improve outcomes |
| Overall representativeness of this study | This phase 1 study was conducted in the USA, with the primary objectives being safety, pharmacokinetics, and laboratory abnormalities of fianlimab and cemiplimab combination treatment for the treatment of solid tumors and lymphoma. The median age of the 78 patients in our study population was in the range of 60–68 years depending on the treatment arm. There were more female patients enrolled in the study than male. Diverse primary cancer types were presented, and the most reported tumor type was colorectal cancer followed by lung cancer. Over 90% of patients received at least one line of prior treatments. The heterogenicity of the study population is typical for this study type and will be mitigated in subsequent studies |

# References

1. Sung H et al. Global Cancer Statistics 2020: GLOBOCAN Estimates of Incidence and Mortality Worldwide for 36 Cancers in 185 Countries. *CA Cancer J Clin* 2021;**71**(3):209-49.
2. National Cancer Institute, Surveillance, Epidemiology and End Results Program. Cancer Stat Facts: Cancer of Any Site. [https://seer.cancer.gov/statfacts/html/all.html. Accessed May 14](https://seer.cancer.gov/statfacts/html/all.html. Accessed%20May%2014), 2024.
